# Supplementary material for: High Incidence of Amoxicillin-Induced Crystal Nephropathy in Patients Receiving High Dose of Intravenous Amoxicillin
Source: J Clin Med. 2020 Jun 27;9(7):2022. doi: 10.3390/jcm9072022 (PMC7409099; doi:10.3390/jcm9072022)
Supplement: Supplementary file 1 [file jcm-09-02022-s001.pdf]

Supplemental Table 1.

	**No AKI (n=285)**	**AKI (73)**	**P**
Males, n (%)	198 (69.5)	46 (63.0)	0.290
Age, years	70.0 (58.5-82.0)	71.0 (64.0-81.5)	0.339
Weigh, Kg	75.0 (63.5-85.6)	77.0 (63.0-90.0)	0.694
BMI, Kg/m^2	26.0 (22.9-30.5)	26.2 (21.5-31.1)	0.818
Hypertension	140 (49.1)	46 (63.0)	**0.034**
Diabetes mellitus	59 (20.7)	23 (31.5)	**0.050**
Length of hospital stay, days	18.0 (12.0-28.0)	27.0 (16.0-42.5)	**<0.001**
Medical admission, n (%)	232 (81.4)	49 (67.1)	**0.008**
Serum creatinine at admission, µmol/L	76.0 (60.0-98.0)	84.0 (61.3-109.0)	0.200
eGFR at admission, $ml/min/1.73m^2$	94.5 (69.3-128.4)	88.3 (59.5-124.6)	0.157
Serum creatinine at discharge, µmol/L	68.0 (57.8-86.3)	92.0 (79.0-149.8)	**<0.001**
eGFR at discharge, $ml/min/1.73m^2$	98.0 (73.2-120.5)	60.8 (39.7-89.0)	**<0.001**
Endocarditis, n (%)	79 (27.7)	31 (42.5)	**0.015**
Hospital mortality, n (%)	15 (5.3)	21 (28.8)	**<0.001**

Supplemental Table 2.

	All (n=42)	AICN (n=16)	Other AKI (n=26)	P*
Males, n (%)	28 (66.7)	7 (43.8)	21 (80.8)	**0.013**
Age, years	75.5 (64.0-82.0)	76.5 (72.0-81.5)	70.0 (63.5-82.0)	0.444
Weight, Kg	79.0 (65.4-93.7)	77.0 (60.0-80.0)	82.0 (66.0-97.0)	0.119
BMI, Kg/m^2	28.6 (23.5-31.9)	27.0 (21.0-30.6)	29.4 (24.8-32.5)	0.334
Hypertension	27 (64.3)	11 (68.7)	16 (61.5)	0.636
Diabetes mellitus	13 (31.0)	5 (31.2)	8 (30.8)	0.974
Medical admission, n (%)	32 (76.2)	14 (87.5)	18 (69.2)	0.177
Serum creatinine at admission, µmol/L	88.0 (61.8-113.5)	92.5 (60.5-114.8)	81.0 (61.5-110.8)	0.786
eGFR at admission, ml/min/1.73m^2	77.0 (50.9-105.7)	73.8 (43.7-107.4)	86.1 (55.0-107.3)	0.393
Serum creatinine at discharge, µmol/L	97.0 (83.0-174.0)	87.0 (86.0-154.0)	123.5 (78.5-283.8)	0.281
eGFR at discharge, ml/min/1.73m^2	51.1 (31.6-80.6)	59.1 (36.7-77.7)	46.3 (20.2-89.7)	0.573
Endocarditis, n (%)	16 (38.1)	6 (37.5)	10 (38.5)	0.950
Hospital mortality, n (%)	12 (28.6)	3 (18.8)	9 (34.6)	0.316

* Comparison between AICN and other AKI groups.

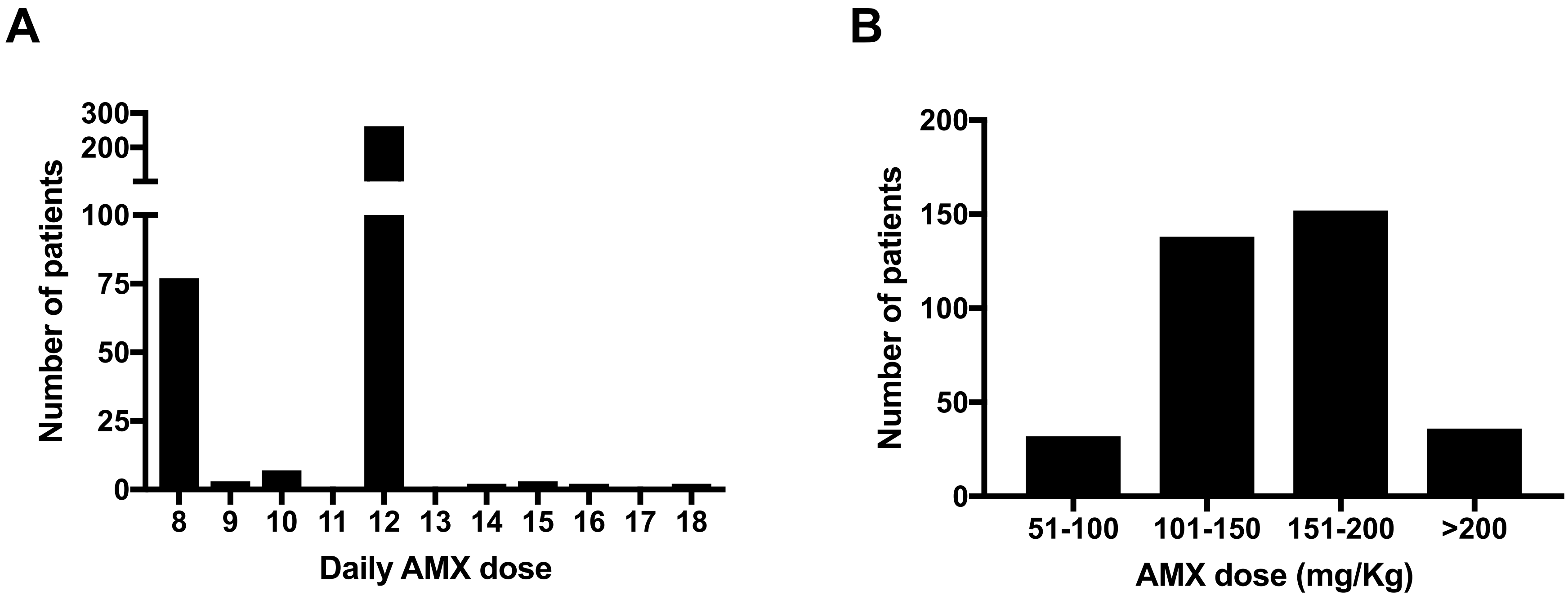
A
Number of patients
300
200
100
75
50
25
0
8
9
10
11
12
13
14
15
16
17
18
Daily AMX dose
B
Number of patients
200
150
100
50
0
51-100
101-150
151-200
>200
AMX dose (mg/Kg)
